# Self-assembled Cubic Boron Nitride Nanodots

**DOI:** 10.1038/s41598-017-04297-1

**Published:** 2017-06-22

**Authors:** Alireza Khanaki, Zhongguang Xu, Hao Tian, Renjing Zheng, Zheng Zuo, Jian-Guo Zheng, Jianlin Liu

**Affiliations:** 1 Quantum Structures Laboratory, Department of Electrical and Computer Engineering, University of California, Riverside, CA 92521 USA; 2 Irvine Materials Research Institute University of California, Irvine, CA 92697-2800 USA

## Abstract

One of the low-dimensional Boron Nitride (BN) forms, namely, cubic-BN (c-BN) nanodots (NDs), offers a variety of novel opportunities in battery, biology, deep ultraviolet light emitting diodes, sensors, filters, and other optoelectronic applications. To date, the attempts towards producing c-BN NDs were mainly performed under extreme high-temperature/high-pressure conditions and resulted in c-BN NDs with micrometer sizes, mixture of different BN phases, and containing process-related impurities/contaminants. To enhance device performance for those applications by taking advantage of size effect, pure, sub-100 nm c-BN NDs are necessary. In this paper, we report self-assembled growth of c-BN NDs on cobalt and nickel substrates by plasma-assisted molecular beam epitaxy. It is found that the nucleation, formation, and morphological properties of c-BN NDs can be closely correlated with the nature of substrate including catalysis effect, lattice-mismatch-induced strain, and roughness, and growth conditions, in particular, growth time and growth temperature. The mean lateral size of c-BN NDs on cobalt scales from 175 nm to 77 nm with the growth time. The growth mechanism of c-BN NDs on metal substrates is concluded to be Volmer-Weber (VW) mode. A simplified two-dimensional numerical modeling shows that the elastic strain energy plays a key role in determining the total formation energy of c-BN NDs on metals.

## Introduction

Boron nitride (BN) is a III-V compound and isoelectronic to the similarly structured carbon lattice, i.e., can possess sp^2^- and sp^3^-bonded phases. This feature leads to a variety of crystalline BN forms including hexagonal (h-BN), rhombohedral (r-BN), turbostratic (t-BN), cubic (c-BN) and wurtzite (w-BN). Among all these crystalline forms, bulk c-BN is most thermodynamically stable, which has excellent physical and chemical properties such as super high hardness (>70 GPa), wide direct band-gap energy (Eg ≈ 6.4 eV), doping ability for both p- and n-type conductivity, very high isotropic thermal conductivity (13 Wcm^−1^K^−1^) along with very low linear thermal expansion (1.2 × 10^−6^ °C^−1^), low dielectric constant (7.1), high breakdown field (~0.7 Vnm^−1^), high-oxidation resistant (>1300 °C), and high structural and chemical stability. These intriguing properties have attracted many researchers to work on synthesis, characterization, and implementation of c-BN bulk and thin film materials for a broad range of mechanical, thermal and optoelectronic applications^1–10^.

Low-dimensional c-BN nanodots (NDs) have their unique applications in addition to those enabled from their bulk counterparts. More specifically, the nanometric size c-BN nanodots with a large surface area can enhance performance of many devices and systems. For example, c-BN NDs can be integrated with lithium metal oxide/phosphide (LiMO_2_/LiMPO_4_) cathode materials used in Li-ion batteries and act as a fast heat dissipater with low thermal expansion to improve their safety features, or as an agent to increase their thermal and chemical stabilities^11^. Also, c-BN NDs can be used as a catalyst support for all applications in which the process requires relatively high temperatures^12^. Furthermore, they are considered as a well-suited candidate for variety of biological applications such as high-contrast bioimaging probe, cell tracking, and gene technology owing to their fast and uniform agglomeration around the targeting material, strong fluorescence properties, and excellent biocompatibility^13^. Nanometric c-BN dots can be also used as a very fine polishing agent because of their size and high hardness^14^. Surface-plasmon-mediated deep ultraviolet ND light emitting diodes, sensors, and electrically insulating/thermally conductive filters in high-power/high-frequency electronics operating in harsh environments are among other examples that may also benefit from c-BN NDs.

Industrial c-BN materials are produced in large quantities as powders with sizes ranging from submicron to millimeters by a high-pressure high-temperature (HPHT) method. However, because of very high growth temperature, in turn, fast growth rate, the size of c-BN particles could not easily reach below submicron^15^. So far, there are very few experimental works for preparation of nanometric c-BN particles or NDs. Among these efforts, phase transformation, laser ablation, and solvothermal reaction have been mostly attempted. Phase transformation of graphite-like (hexagonal) or amorphous BN precursors usually requires extremely high pressure of several tens GPa and high temperature of 1300~1700 °C^16, 17^. Laser ablation technique also uses hexagonal BN precursors, which requires a high-power laser to provide locally high pressure/temperature for c-BN NDs growth^18^. Chemical routes can also generate a large amount of c-BN materials, however, controlling the size, morphology, phase and purity of as-grown NDs is difficult thanks to low reaction temperature^19, 20^. Aside from having to use extreme growth conditions, such synthesis approaches often lead to the final products with either a mixture of different BN phases, e.g. h-BN/c-BN, or containing process-related impurities/contaminants^16–20^.

In contrast, self-assembled growth of NDs has been identified as an important nanofabrication process in which the building blocks spontaneously organize into random and/or ordered distributions by thermodynamic and other constraints^21^. However, in order to successfully exploit NDs self-assembly in technological applications and to ensure effective scale-up, a high level of control using ultra high vacuum chemical vapor deposition (CVD) or molecular beam epitaxy (MBE) is desirable. As a versatile tool, MBE has natural advantages in high-quality materials growth including thin films and self-assembled NDs because of its ultra-high vacuum environment and instant introduction and control of multiple sources^22–28^. Therefore, the direct growth of c-BN NDs by MBE offers reduced complexity of fabrication procedure as well as a great degree of quality, purity, and reproducibility. Here we report MBE growth and characterization of self-assembled c-BN NDs on cobalt (Co) and nickel (Ni) metal substrates. A simplified numerical model is also introduced to simulate the formation energy behaviour of c-BN NDs on metal substrates.

## Results

### Self-assembled growth of c-BN NDs on Co substrate

**Figure 1:**
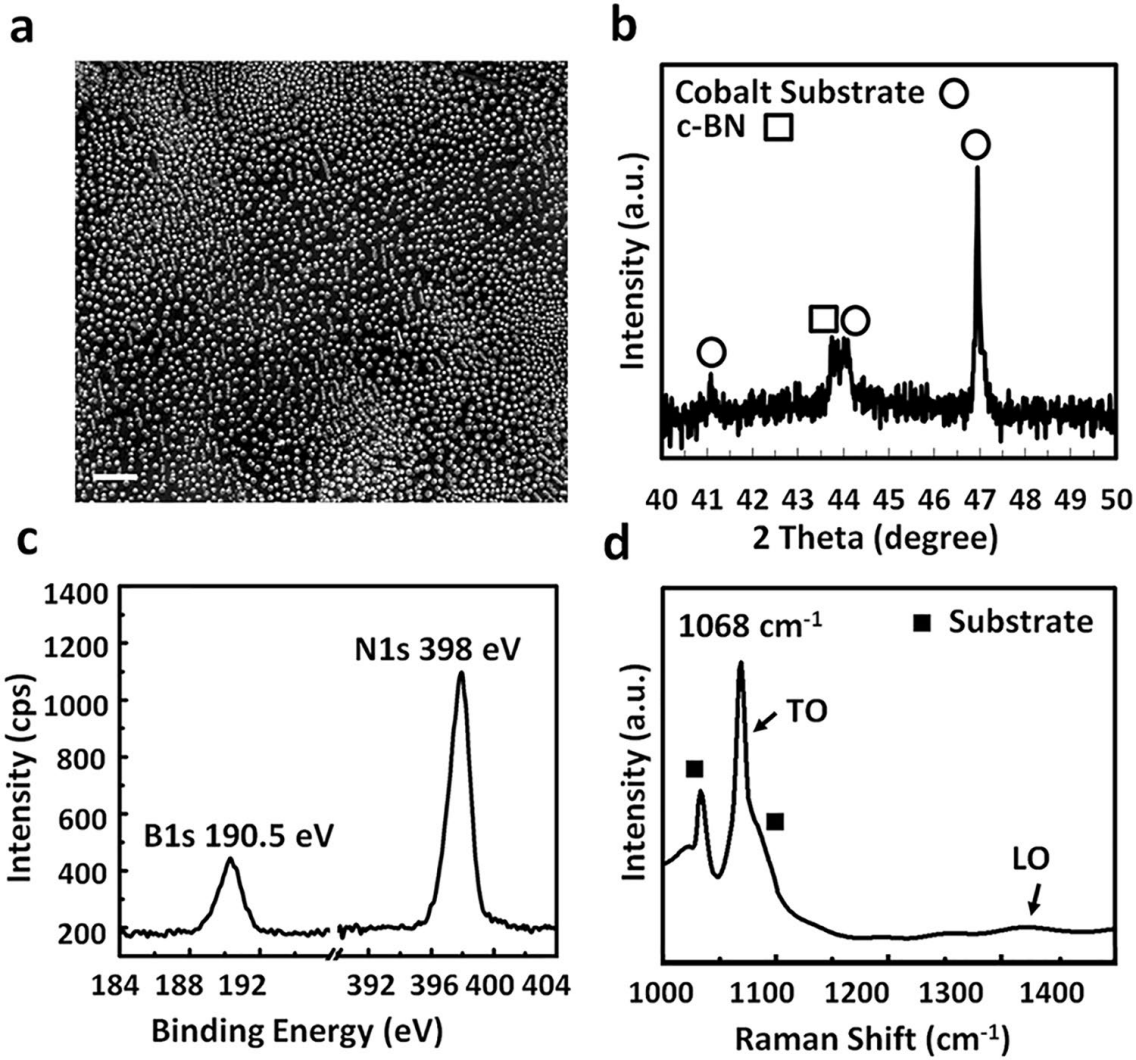
Characterization of self-assembled c-BN NDs grown on Co foil substrate by plasma-assisted MBE. The sample was grown at 900 °C for 10 minutes. (**a**) SEM image of the sample showing a relatively uniform distribution of NDs over a large area. The average NDs lateral size and density are estimated to be ~87 nm and ~4.08 × 10^9^ cm^−2^, respectively. The scale bar is 1 μm. (**b**) Short-range X-ray diffraction pattern of c-BN NDs on Co foil substrate showing a dominant peak at 43.89° corresponding to (111) crystal plane diffraction of c-BN NDs with the fcc structure. (**c**) B1s and N1s XPS signals of NDs. (**d**) Raman spectrum of c-BN NDs showing an evident TO phonon peak located at 1068 cm^−1^.

Figure 1(a) shows an SEM image of a c-BN NDs sample, which was grown on Co foil substrate at 900 °C for a duration of 10 minutes, showing a relatively uniform distribution of NDs over a large area of the sample. Using ImageJ software, the density of NDs was estimated to be ~4.08 × 10^9^ cm^−2^ (see Supplementary, Fig. S1). The lateral size of NDs was also estimated to be 80~90 nm. Figure 1(b) shows X-ray diffraction pattern of the c-BN NDs sample. The dominant peak at 43.89° was assigned to (111) crystal plane diffraction of c-BN NDs with the fcc structure according to JCPDS#025-1033. Short-range (2θ: 40~50°) survey was chosen for Fig. 1(b) to resolve the peak deconvolution for c-BN (111) and Co (002) occurred near 44° diffraction angle. Full-range XRD pattern (2θ: 20~90°) of the sample is also shown in Fig. S2 (see Supplementary). According to the full-range scan, no other diffraction of BN related materials can be observed, suggesting that NDs are dominantly cubic in structure^16^. Due to the much lower peak intensity compared to those from the substrate, the second and third order diffraction peaks of c-BN NDs are not detected in the spectrum. The calculated unit cell parameter from the XRD result of c-BN NDs is 3.57 Å. A slight change from its bulk number of 3.61 Å suggests that some strain still exists after the formation of the dots^29^. It is worth mentioning that the lattice of c-BN NDs may be distorted unevenly in the in-plane and cross-plane directions since the θ/2θ peak only gives information about (111) plane distance. Furthermore, as the self-assembly formation of dots represents a process of strain relaxation due to lattice mismatch between the dots and substrate, the degree of strain relaxation may also vary from dots with different sizes and shapes, and from near the substrate-NDs interface to the upper part within the same dot, as shown in TEM imaging later. Figure 1(c) shows XPS spectrum of B1s and N1s signals. B1s and N1s exhibit an energy position at 190.5 eV and 398.0 eV, respectively, which are typical characteristics of c-BN material^30^. By using sensitivity factors from the instrument manufacturer and calculating atomic % of each atom, the B/N ratio is 1.01, suggesting an almost equal composition of boron and nitrogen elements.

Figure 1(d) shows a Raman spectrum of the c-BN NDs sample. A peak at 1068 cm^−1^ is evident, which shows a large shift of 12 cm^−1^ towards a higher wavenumber compared to the characteristic transverse optical (TO) phonon mode of c-BN bulk material (1056 cm^−1^)^31^. This could be due to the strain effect induced by lattice mismatch between the Co substrate and c-BN NDs and/or internal NDs crystal defects^31^. The intensity of the longitudinal optical (LO) phonon mode at about 1306 cm^−1^ is much weaker than that of the TO phonon mode, which could be due to the size effects from c-BN NDs or internal lattice defects^32^. Also, according to the Raman selection rule, the observation of TO and LO phonon modes indicates that the c-BN NDs growth has dominantly occurred along <111> direction, which is consistent with our XRD results (Fig. 1(b))^33^. No h-BN phonon modes (e.g., E_2g_ at about 1366 cm^−1^) are observed as well. The full width at half maximum (FWHM) of the TO phonon peak is 10 cm^−1^, which is twice as large as that of a single crystal c-BN material having a high quality and a size of 1~2 mm obtained by a high-pressure method^34^. The difference could be explained by the small and non-uniform size effects on the FWHM of the phonons in the NDs^35^. A peak at 1034 cm^−1^ and its shoulder at around 1086 cm^−1^ in the Raman spectrum are mainly caused by the post-growth Co surface oxidation and subsequent formation of CoO, Co(OH)_3_, CoO(OH), and Co_3_O_4_ (see Supplementary, Fig. S3)^36–38^. Other materials such as boron oxide (B_2_O_3_)^39^, boric acid (H_3_BO_3_)^40^, boron carbide (B_4_C)^41^, carbon nitride (CN)^42^, and cobalt boride (CoB)^43^ do not exist in our c-BN NDs based on their Raman, XRD, and XPS characteristics, suggesting the formation of pure c-BN phase in this synthesis method.

**Figure 2:**
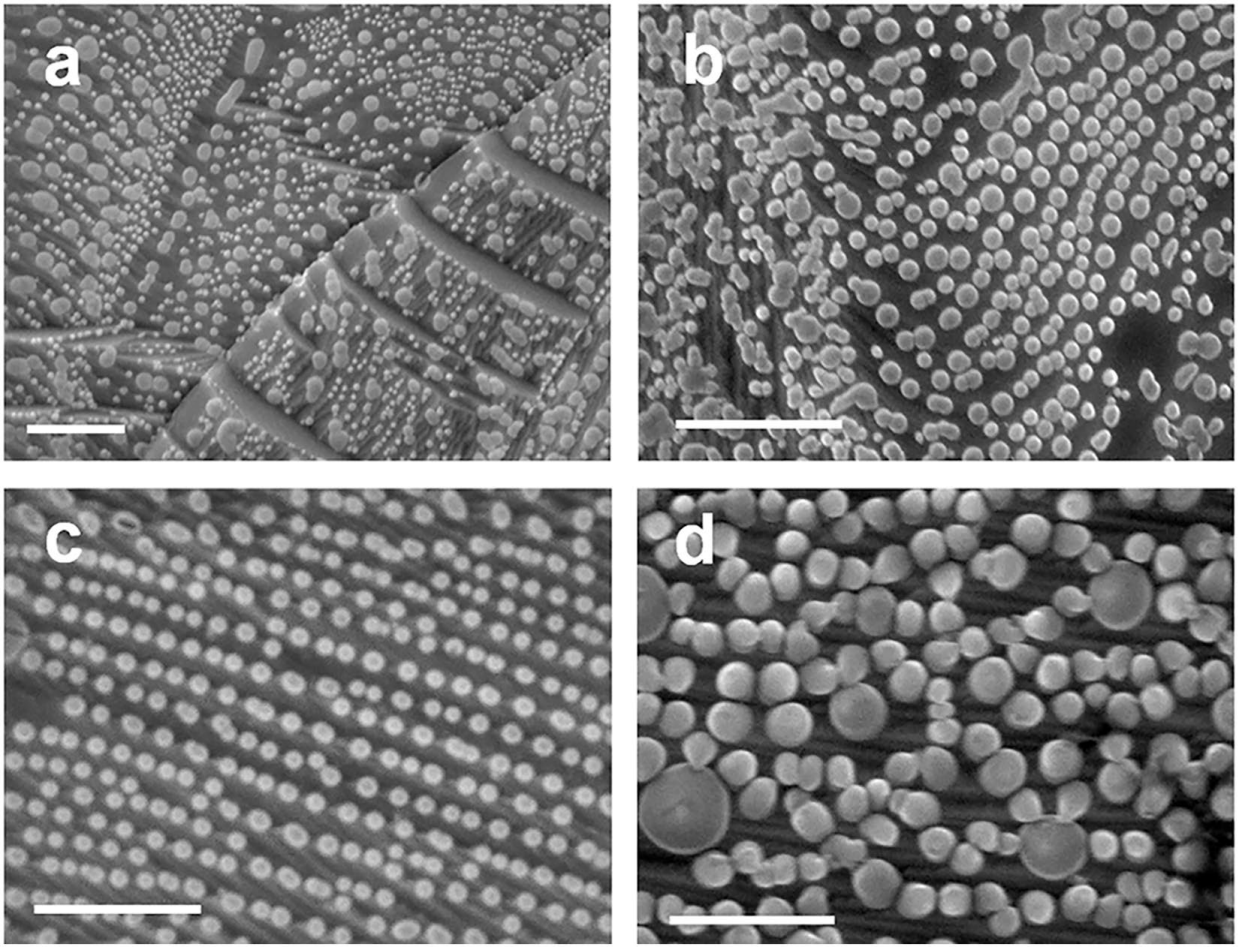
Morphology evolution of the c-BN NDs as a function of growth time. SEM images of samples grown at (**a**) 10 seconds, (**b**) 30 seconds, (**c**) 5 minutes, and (**d**) 45 minutes. All the samples were grown at the same growth temperature of 900 °C and all the scale bars are 1 µm.

Figure 2(a–d) show SEM images of c-BN NDs samples grown at different growth time of 10 seconds, 30 seconds, 5 minutes, and 45 minutes, respectively. All the samples were grown at 900 °C. Therefore, together with the 10-minute sample discussed earlier, we have total five samples to study the growth time dependence. According to Fig. 2(a) and (b), even at a short growth time of 10 or 30 seconds, high-density NDs have been formed on the Co foil surface, suggesting that the growth rate should have been fast. The fast growth rate of NDs within a short period of time can be due to the catalytic effect of Co substrate, which reduces the NDs growth formation energy^44–46^. In addition, the rough surface of the Co substrate (Rq ~ 187 nm) provides a great number of preferential nucleation sites for NDs growth^47^. As a result, NDs notably increase in size as the growth time increases within this short growth time (Fig. 2(a) and (b)). However, as the growth time further increases (up to 45 minutes), the NDs growth rate reduces and the NDs become more uniform in size and shape (Fig. 2(c) and (d)). This is reasonable since it is less thermodynamically favorable for incoming boron and nitrogen atoms to grow upon previously grown c-BN NDs compared to that of fresh Co foil surface thanks to different formation energy on these surfaces^48, 49^. In addition, it has been theoretically shown that the surface free energy barrier is the largest at the edges of the NDs due to NDs surface strain, and decays as the adatoms move away from them, further suggesting that the adatoms tend to form new NDs instead of accumulating with previously grown ones^49^. As the growth time lapses, the substrate surface is eventually covered with dense NDs and simultaneously run out of preferential nucleation sites. Further introduction of sources onto the substrate would lead to the accumulation of adatoms on existing NDs. During this period, two competing processes may co-exist for those relatively small NDs: higher-strained, smaller NDs grow faster than less-strained larger ones in order to reduce their larger surface strain and total free energy^49, 50^. On the other hand, boron and nitrogen atoms may leave very small or irregular-shaped NDs to settle into larger NDs^51, 52^. The competing processes eventually bring about more uniformity in size with time. It should be also noted that apparent chain-like alignment of NDs is observed in the SEM images. This is related to the substrate roughness effect, which is discussed later^53^.

**Figure 3:**
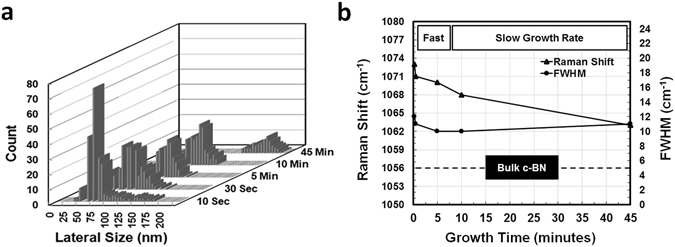
Growth time-dependent features analysis. (**a**) Graphical representation of c-BN NDs on Co lateral size distributions by histogram; (**b**) Raman shift and FWHM of TO phonon peak of c-BN NDs versus growth time. Raman shift evolves from 1073 to 1063 cm^−1^. The size dependence of FWHM of the TO phonon peak is weak in a range of 10~12 cm^−1^. The dashed line in (**b**) shows the position for characteristic TO phonon peak and FWHM of bulk c-BN.

Figure 3(a) shows histogram plot of NDs lateral size distributions as a function of growth time using ImageJ software (see Supplementary, Fig. S1). The number of NDs and their lateral size were approximated for an area of 4 μm^2^. It is worth mentioning that the effort to extract vertical height information of these dots by AFM was halted as a result of quite rough Co foil substrate surface. According to Fig. 3(a), the distribution of ND sizes narrows from 30~175 nm (mean: 77.6 nm, standard deviation: 38.3 nm) to 30~155 nm (mean: 93 nm, standard deviation: 23.8 nm), 50~142 nm (mean: 102.5 nm, standard deviation: 16 nm), 62~190 nm (mean: 112.7 nm, standard deviation: 19.2 nm), and finally 140~210 nm (mean: 174.4 nm, standard deviation: 18.6 nm) as the growth time increases from 10 seconds to 30 seconds, 5 minutes, 10 minutes and finally to 45 minutes. Moreover, since the area of survey for each sample is constant (4 μm^2^), the number of NDs becomes less as the growth time progresses, suggesting that NDs become larger and their density reduces. Figure S4 (see Supplementary) shows Raman spectra for all five samples and Fig. 3(b) shows Raman shift and FWHM of TO phonon peak of ND sample as a function of growth time. Raman characteristic peak blue-shifts from 1073 to 1063 cm^−1^ as the NDs enlarge with the growth time. Interestingly, the rate change in Raman peak wavenumber is also in accordance with the observed trend for changes in the growth rate of NDs in the SEM images (Figs 1(a) and 2). However, the FWHM of the TO phonon peak in a range of 10~12 cm^−1^ does not have a clear dependence of the size of the dots. It should be noted that the intensity of LO phonon modes decreases as the decrease of the growth time, which may be due to relaxation of Raman selection rules for smaller c-BN NDs^32, 33^.

### Self-assembled growth of c-BN NDs on Ni substrate

**Figure 4:**
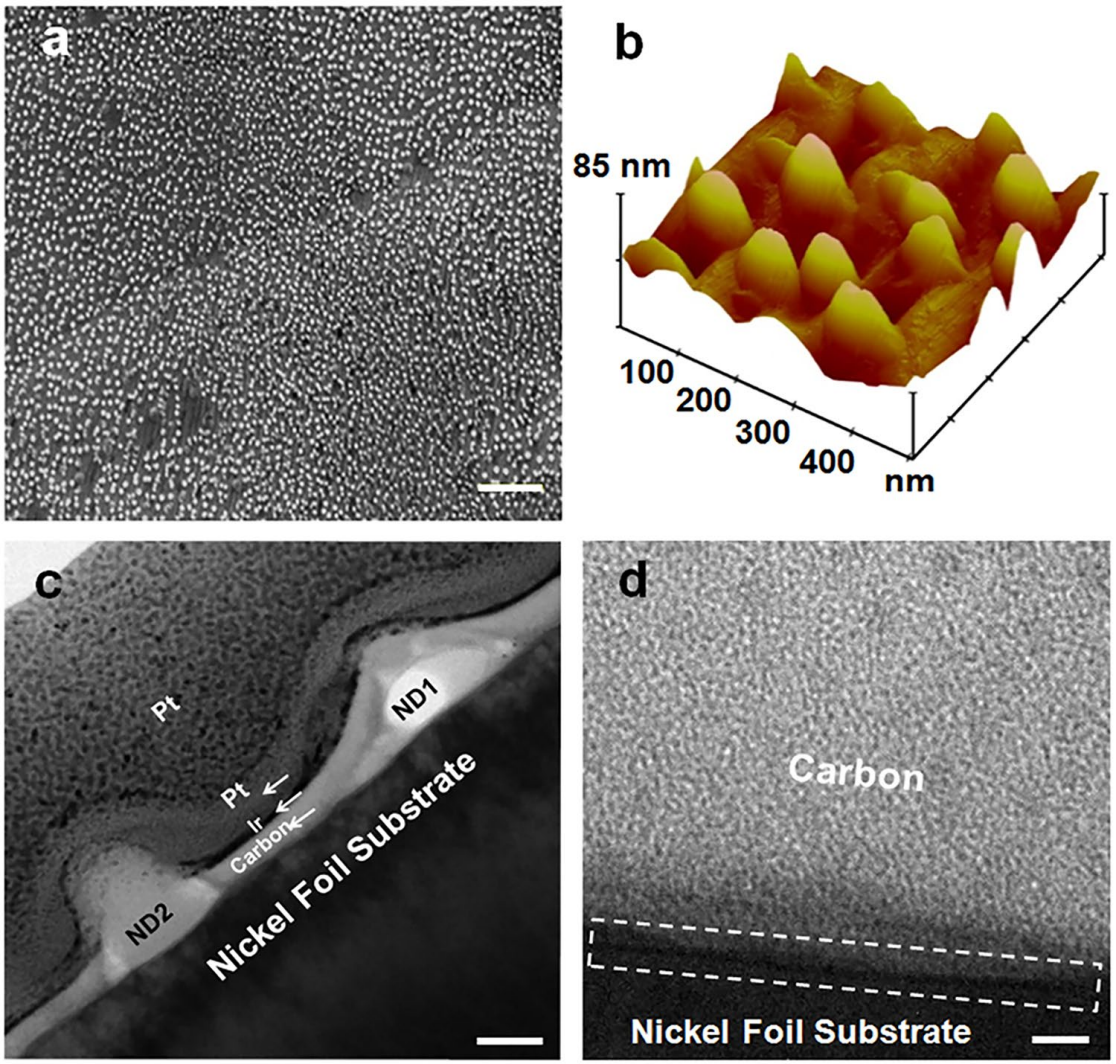
Characterization of c-BN NDs on Ni foil substrate. The sample was grown at 900 °C for 10 minutes. (**a**) SEM image of the sample. The NDs are uniformly distributed over a large area. The density of NDs is estimated to be ~2.3 × 10^9^ cm^−2^. The scale bar is 1 μm. (**b**) AFM image of the sample, c-BN NDs have average lateral and vertical sizes of ~95 and ~45 nm, respectively. (**c**) Cross-sectional bright field TEM image of the sample. Two c-BN NDs are resolved in the image. (**d**) Higher resolution bright field TEM image of the sample. The dashed-line rectangle shows the interface between the carbon coating layer and Ni foil substrate between the two NDs. No c-BN wetting layer underneath or near the NDs area can be observed, indicating VW growth mode.

In order to study different substrate effect on the formation of c-BN NDs, Ni foil was also used. Because of its similarity with Co in terms of catalytic effect^54^ and lattice mismatch with c-BN, it is expected that c-BN NDs can also be formed on Ni substrate. Figure 4(a) shows an SEM image of a c-BN NDs sample grown at 900 °C for 10 minutes. X-ray diffraction, XPS, and Raman spectroscopy characterization of the sample indicates that these NDs are c-BN (see Supplementary, Fig. S5). As seen from the SEM image, a relatively uniform distribution of c-BN NDs is achieved over a large area of the Ni substrate. The density of NDs is estimated to be around ~2.3 × 10^9^ cm^−2^, which is lower than that on Co foil sample under the same growth condition (Fig. 1(a)). This might be due to the smoother surface of Ni foil, in turn, less preferential nucleation sites (see Supplementary, Fig. S6). Using AFM characterizations (Fig. 4(b)), the average lateral and vertical sizes of c-BN NDs were estimated to be ~95 and ~40 nm, respectively.

Figure 4(c) shows a bright field cross-sectional TEM image of the sample. Two c-BN NDs are evident within the imaging area. Figure 4(d) shows a higher resolution bright field TEM image of the sample area between the two NDs. A dashed-line rectangle in Fig. 4(d) is used to guide the eyes on the interface between the carbon coating layer and Ni foil substrate. No c-BN epilayer/wetting layer underneath or between the NDs can be observed, indicating that the c-BN ND growth undergoes Volmer-Weber (VW) growth mode^49^. This is reasonable because the lattice mismatch between c-BN and Co or Ni substrates prevents the formation of strained two-dimensional BN wetting layer at the early stage of the growth. Instead, small amount of deposited material is accumulated so as to form NDs directly on the substrate. In addition, insolubility of BN in Ni, Co and related alloys at high temperatures causes the boron and nitrogen atoms to more strongly bond to one another than to the underlying substrate, which encourages VW growth mode, as in the case of the growth of many metals on insulator substrates or vice versa^55–57^. The measured contact angle of 135° (i.e., >90°) for c-BN NDs on Ni substrate can be another indication for the presence of strain at the NDs/substrate interface and subsequent VW growth mechanism (see Supplementary, Fig. S7(a))^58, 59^. Moreover, according to Fig. S7(b) (see Supplementary), higher resolution bright field TEM images at the interface proximity between the c-BN ND and Ni substrate (dashed-line rectangle) show a darker contrast compared to the surrounding area of ND, implying different atomic structures, lattice spacing, or orientations at such locations which could be another sign of strain^60, 61^. It is worth noting that the amount of lattice mismatch between c-BN structure (zinc blende) and underneath metal substrate (hcp Co or fcc Ni) can vary significantly depending on the ND and substrate surface crystal orientations. Our Co and Ni foil substrates are polycrystalline in nature (see Supplementary, Figs S2 and S5), and even within a single grain, their rough surfaces can expose different facets with different orientations for the growth of c-BN NDs. For example, the lattice mismatch between the c-BN (111) and Co (001) would be ~2% (α_c-BN(111)_ = 2.55 Å, α_Co(001)_ = 2.50 Å), while for other possible crystal orientations such as c-BN (001) on Co (001), it can be as large as ~44.4% (α_c-BN(001)_ = 3.61 Å) (see Supplementary, Fig. S8).

### C-BN NDs Formation Energy Modeling

**Figure 5:**
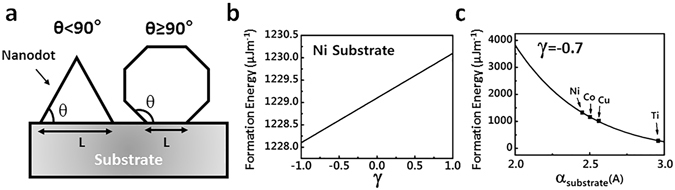
Formation energy of c-BN ND on metal substrates. (**a**) 2D model of a strained self-assembled c-BN ND growth on a flat metal substrate via VW growth mode and different contact angles. (**b**) Total formation energy of a c-BN ND with the contact angle of 135° grown on flat Ni (111) substrate as a function of γ. (**c**) Total formation energy of a c-BN ND with the contact angle of 135° and γ fixed at −0.7 as function of substrate unit cell parameter (α_substrate_). Unit cell parameters (along (111) crystal plane) of different transition metal substrates have been tagged in panel c.

To further understand the formation of c-BN ND nucleation on metal substrates, we have carried out numerical modeling of the formation energy. The formation energy can be written as:

1 $${{\rm{E}}}_{{\rm{F}}}={{\rm{E}}}_{\mathrm{surf} \mbox{-} \mathrm{ND}}+{{\rm{E}}}_{{\rm{int}}}-{{\rm{E}}}_{\mathrm{surf} \mbox{-} \mathrm{sub}}+{{\rm{E}}}_{{\rm{mismatch}}}$$

where E_F_ is the total formation energy of ND with a unit of (Jm^−1^), E_surf-ND_ is the surface energy of ND, E_int_ is the interface energy of the interface between two materials, E_surf-sub_ is the surface energy of the substrate covered by ND, and E_mismatch_ is the elastic strain energy stored in ND with a unit of (Jm^−1^). The first three terms are dominating factors in the determination of strainless NDs on a substrate, for example, Si dots on SiO_2_ substrate^53^. The last term is included to accommodate the elastic energy strain as a result of lattice mismatch between NDs and underneath metal substrate^62, 63^. It should be noted that the formulation of formation energy in Equation (1) does not include contribution from catalytic effect because it would be different for different metal substrates, differs significantly with growth conditions such as growth temperature. Equation (1) can be expressed as the following equation:

2 $${E}_{F}=L{\gamma }_{ND}(\text{sec}\,{\rm{\theta }}+\frac{{\gamma }_{\mathrm{int}}-{\gamma }_{sub}}{{\gamma }_{ND}})+\frac{1}{2}SE\varepsilon .$$

where γ_ND_, γ_int_, and γ_sub_ are the surface energy density of ND, interface, and substrate, respectively. L, θ, and S are ND lateral size, contact angle, and the area in two-dimensional (2D) model, respectively. $\gamma =(\frac{{\gamma }_{\mathrm{int}}-{\gamma }_{{\rm{sub}}}}{{\gamma }_{{\rm{ND}}}})$ is defined as a non-dimensional system parameter, which varies between -1 and 1 based on the contact angle (θ) and according to the Young equation^63^. Contact angle also relates to ND size and the nature of the two materials^64–66^ E is Young module of ND, and $\varepsilon =(\frac{{\alpha }_{{\rm{ND}}}-{\alpha }_{{\rm{sub}}}}{{\alpha }_{{\rm{sub}}}})$ is strain caused by lattice mismatch between the ND and metal substrate. α_ND_ and α_sub_ are the lattice constant of ND and unit cell parameter of substrate, respectively. Since it is possible that a nanodot adopts a contact angle upon its nucleation (e.g. θ ≤ 90°) and increases it during the growth (e.g. θ ≥ 90°), different equations must be used to simulate the formation energy of NDs based on their contact angles and shapes^58, 59^. For a contact angle of below 90°, NDs can be simulated as triangles (Fig. 5(a)), and the total formation energy can be written as:

3 $$\begin{array}{rcl}{E}_{F} & = & {\gamma }_{ND}(\sec \,\theta +\frac{{\gamma }_{\mathrm{int}}-{\gamma }_{sub}}{{\gamma }_{ND}}){S}^{\frac{1}{2}}{(\tan \theta )}^{-\frac{1}{2}}+\frac{1}{2}SE{\varepsilon }^{2}\\ \theta  &  <  & 90^\circ \end{array}$$

while for a contact angle of above 90°, NDs can be simulated as regular polygons with different number of sides (same length), where the number of the sides are determined by a type of polygon which has the closest inner angle to that of ND contact angle (Fig. 5(a)). Therefore, the total formation energy for a ND with a contact angle of above 90°can be written as:

4 $$\begin{array}{rcl}{E}_{F} & = & 2{\gamma }_{{\rm{ND}}}(\sec \,{\rm{\theta }}+\frac{{\gamma }_{{\rm{int}}}-{\gamma }_{{\rm{sub}}}}{{\gamma }_{{\rm{ND}}}}){{\rm{S}}}^{\frac{1}{2}}\,\tan \,{(\frac{\theta }{{\rm{n}}-2})}^{\frac{1}{2}}{{\rm{n}}}^{-\frac{1}{2}}+\frac{1}{2}{\rm{SE}}{\varepsilon }^{2}\\ {\rm{n}} & \ge  & 4\\ \theta  & \ge  & 90^\circ \end{array}$$

where n is the number of the sides for the polygon.

To run the simulation, we assumed a c-BN ND (α_ND(001)_ = 3.57 Å) was formed on top of an idealized flat surface of a metal substrate with (111) crystal orientation and a fixed contact angle of θ = 135° (see Supplementary, Fig. S7(a)). The best polygon that can represent a ND with θ = 135° would be an octagon (n = 8) with inner angle of 135°, and hence, Equation (4) can be used to calculate the ND formation energy. Here the values are chosen as a representative case just to show the overall trend on ND nucleation behavior. The surface energy density and Young module for c-BN NDs are ~40 mJm^−2^ and ~500 GPa, respectively^67, 68^. Figure 5(b) shows the formation energy of c-BN NDs on Ni substrate (α_Ni(111)_ = 2.48 Å) as a function of γ. The larger the γ is, the higher the formation energy is, and hence, the harder it is to form c-BN ND. It should be also noted that the formation energy is linearly proportional to γ, however, the alterations are not significant. In contrast, when γ is fixed (e.g., γ = −0.7), the formation energy changes dramatically and nonlinearly as the substrate unit cell parameter (α_sub_) changes within a range of 2~3 Å, as can be seen in Fig. 5(c). Similar behavior is observed for various γ and lattice parameters (see Supplementary, Fig. S9). These results suggest that the elastic strain energy (E_mismatch_) plays a major role in determining the total formation energy of strained c-BN NDs on metal substrate via VW growth mode. According to our calculation, this behavior is mainly due to the lattice mismatch between the c-BN NDs and underneath metal substrate, and the large value of Young module for c-BN material (~500 GPa). Although the growth of c-BN NDs has been demonstrated on Co and Ni substrates here, based on our calculations (Fig. 5(c)), we hypothesize that it should be possible to grow c-BN NDs on other similar transition metal substrates including titanium (Ti), copper (Cu), etc.

Finally, we discuss the mechanism for seemingly chain-like alignment of NDs on the surfaces as seen from the SEM images in Fig. 2. The Co foil substrates were not ideally flat with ridges and valleys across their surfaces (see Supplementary, Fig. S6). If we take the substrate rough surface morphology effect into account, for example, at concave surfaces where the atomic inter-distances of underneath metal substrate are relatively smaller compared to that of flat substrate surfaces, the lattice mismatch between the substrate and c-BN ND would be less, and hence, the formation energy would be much lower. Therefore, the concave substrate surfaces can act as preferential places for nucleation of c-BN NDs, leading to the chain-like alignment of NDs along the valleys. Using a similar analysis, we can reach an opposite situation for the formation of c-BN NDs on convex substrate surfaces.

## Conclusion

We report self-assembled growth of c-BN NDs on Co and Ni metal substrates by plasma-assisted MBE. The cubic structure was confirmed by detecting the c-BN characteristic TO phonon mode in Raman spectra, (111) crystal plane diffraction peak in XRD pattern, and B1s and N1s signals in XPS results. The time evolution growth of c-BN NDs was observed using SEM and AFM characterizations, and the changes in their size distribution, alignment, density, and internal strain were carefully studied. It was concluded that the substrate (i.e., catalysis effect, lattice-mismatch-induced strain, and roughness), together with the growth conditions (i.e., the growth time and growth temperature) play a key role in the nucleation, formation, and morphological changes of c-BN NDs. The c-BN NDs with a density of ~10^9^ cm^−2^ scale from 175 nm to 77 nm with the growth time. In addition, VW growth mode is responsible for the formation of c-BN NDs and the elastic strain energy is the dominant factor in determining the total formation energy of c-BN NDs on metal substrates. Self-assembly can lead to high-quality, pure, and ultrasmall c-BN NDs in the nanometer range that conventional high-temperature high-pressure methods were not able to achieve, therefore this research paves a way to develop this new form of c-BN materials for a variety of novel applications such as catalysis, battery, biology, deep ultraviolet sensor, and optoelectronic technologies.

## Methods

### Substrate Preparation

Commercial cobalt (Co) foil (Alfa Aesar, 0.1 mm thick, 99.95% purity) and nickel (Ni) foil (Alfa Aesar, 0.025 mm thick, 99.5% purity) were used as substrates. The measured roughness for as-received Co and Ni foils are 187 and 140 nm, respectively (see Supplementary, Fig. S6). The foils were cut into 1 cm × 1 cm pieces and etched by diluted HCl solution (5%) for 1 minute to remove the native oxides. Acetone, isopropyl alcohol (IPA), and deionized water (DI) were used to clean the substrates. After blown dry using a nitrogen gun, these substrates were immediately loaded onto substrate holders and transferred to the growth chamber. A Knudsen effusion cell filled with B_2_O_3_ powder (Alfa Aesar, 99.999%) was used as the boron source. An electron cyclotron resonance (ECR) system was used to form nitrogen gas plasma (Airgas, 99.9999%) as the nitrogen source. The nitrogen source was tuned by either nitrogen gas flow meter or ECR magnetron current.

### C-BN NDs Growth

For a typical growth, the substrate was firstly annealed at 900 °C under a hydrogen flow of 10 sccm for a duration of 15 minutes. At the end of substrate surface treatment, the hydrogen gas flow rate was reduced to 6 sccm and c-BN NDs growth was started. Boron cell temperature was ramped to 1100 °C right before the growth. Nitrogen flow rate was 10 sccm and the ECR current was set at 60 mA. The growth time varied between 10 seconds and 45 minutes and the growth temperature was 900 °C for all samples. At the end of growth, the substrate temperature was cooled towards ambient temperature. The substrate heating/cooling rate was ~10 °C/min.

### C-BN NDs Characterization

Raman characterizations were performed using a HORIBA LabRam system equipped with a 60-mW 532-nm green laser. Scanning electron microscopy (SEM) images were acquired using a XL30-FEG system. Atomic force microscopy (AFM) images were obtained using a Veeco AFM D3100 system. Size distribution and density of c-BN NDs samples grown on Co foil were obtained using ImageJ software. X-ray photoelectron spectroscopy (XPS) was carried out using a Kratos AXIS ULTRA XPS system equipped with an Al Kα monochromatic X-ray source and a 165-mm mean radius electron energy hemispherical analyzer. X-ray diffraction (XRD) (Philips, PW1730) was performed at θ–2θ configuration and using Cu Kα radiation (λ = 1.5405 Å, 40 kV, 40 mA). Transmission electron microscopy (TEM) images were acquired using a FEI/Philips CM-30 TEM. Cross-sectional TEM samples were prepared using the focused ion beam technique. C-BN NDs sample was originally covered by a carbon layer of 26 nm, followed by an Ir layer of about 6 nm, and further protected by electron-beam and ion-beam deposited Pt layers of 41 nm and 1.5 μm, respectively.

## Electronic supplementary material

Supplementary Information
