# Supplementary material for: Self-assembled Cubic Boron Nitride Nanodots
Source: Sci Rep. 2017 Jun 22;7:4087. doi: 10.1038/s41598-017-04297-1 (PMC5481407; doi:10.1038/s41598-017-04297-1)
Supplement: Supplementary file 1 — Supplementary Information [file 41598_2017_4297_MOESM1_ESM.pdf]

Supplementary Information

Self-assembled Cubic Boron Nitride Nanodots

Alireza Khanaki,[1†] Zhongguang Xu,[1†] Hao Tian,[1] Renjing Zheng,[1] Zheng Zuo,[1] Jian-Guo Zheng,[2] Jianlin Liu[1*]

[1]Quantum Structures Laboratory, Department of Electrical and Computer Engineering, University of California, Riverside, CA 92521, USA.
[2]Irvine Materials Research Institute University of California, Irvine, CA 92697-2800, USA.
[†]These authors contributed equally to this work.
[*]Correspondence and requests for materials should be addressed to Jianlin Liu (email: jianlin@ece.ucr.edu, Tel: 1-9518277131, Fax: 1-9518272425)

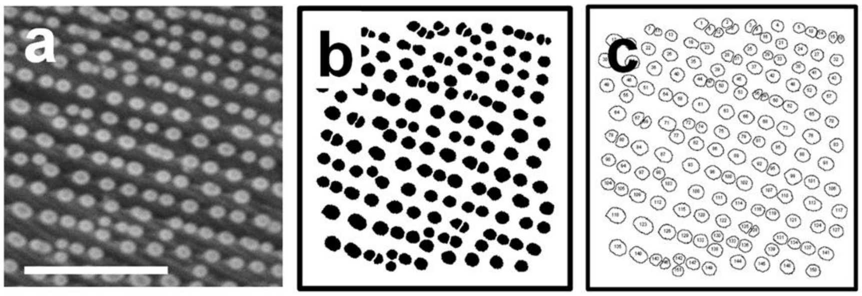

Figure S1. An example of size distribution estimation using ImageJ software. First, an area of 4 μm^2 from an SEM image was selected for each sample. (a) shows the selected area for the sample grown on Co foil at a growth temperature of 900 ºC for 5 minutes. (b) By applying a black/white contrast threshold, NDs were defined as circular features, which are outlined with black circles and accordingly numbered, as shown in (c). Subsequently, the software calculated the area for each ND and the total number of NDs. Finally, their diameter (lateral size) as well as their density were estimated. The scale bar in (a) is 1 μm.

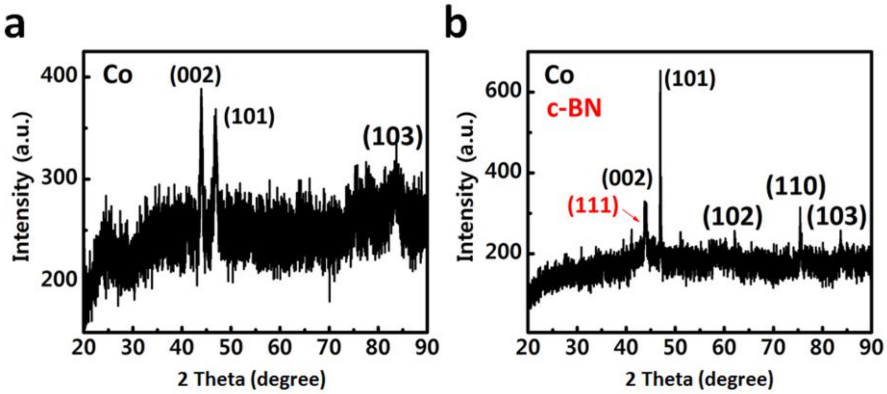

Figure S2. Full range XRD patterns. XRD spectra of (a) as-received Co substrate, and (b) the c-BN NDs grown on Co substrate at 900 °C for a duration of 10 minutes**.** Except a peak at about 44° (see Fig. 1(b) for a better visualization), no other diffraction of BN related materials can be observed in the entire pattern (2θ: 20-90°), suggesting that NDs possess dominantly cubic structure. All other peaks were assigned to diffractions from polycrystalline Co substrate with the hexagonal structure according to JCPDS# 01-071-4239.

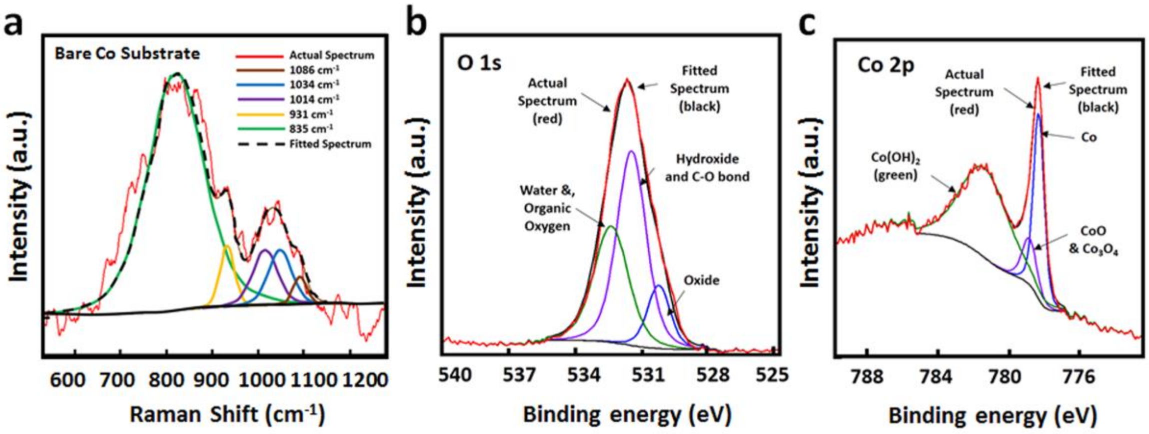

Figure S3. Raman and XPS analyses of the Co substrate. (a) Raman spectrum of the bare Co substrate fitted into multiple peaks. XPS signals of (b) O1s and (c) Co2p for c-BN NDs sample grown on Co substrate at 900 °C for 10 minutes.

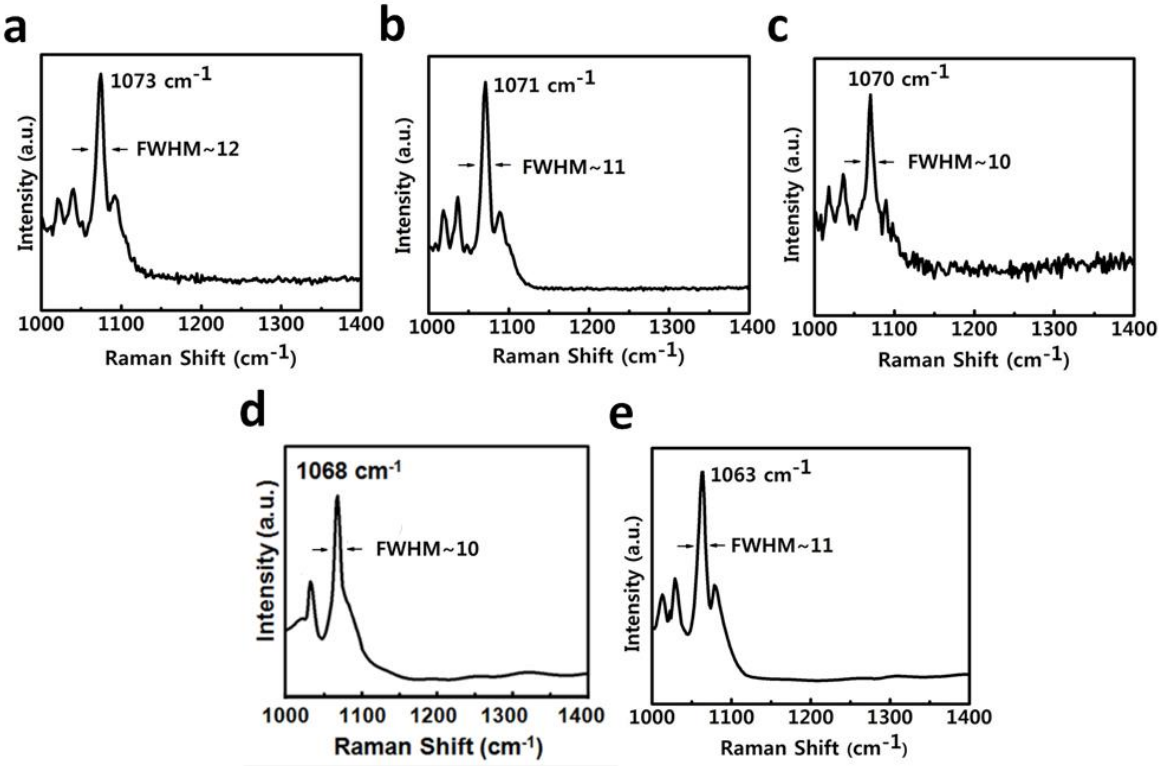

Figure S4. Raman spectra of c-BN NDs on Co substrate. Samples grown at 900 °C for (a) 10 seconds, (b) 30 seconds, (c) 5 minutes, (d) 10 minutes, and (e) 45 minutes.

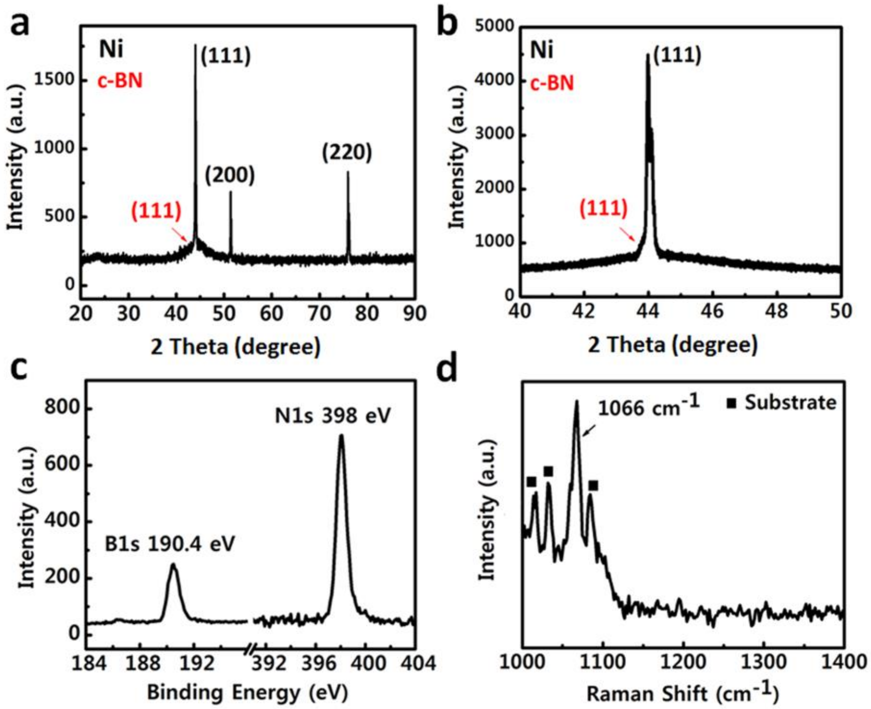

Figure S5. Characterizations of self-assembled c-BN NDs on Ni foil substrate by plasma-assisted MBE. The sample was grown at 900 °C for 10 minutes. (a) Long range and (b) short range X-ray diffraction pattern of c-BN NDs on Ni substrate showing a shoulder at 43.8° near the (111) crystal plane diffraction of Ni substrate. Other diffractions were assigned to the polycrystalline Ni substrate with a fcc structure according to JCPDS# 00-004-0850. (c) B1s and N1s XPS signals of NDs with the B/N ratio of ~1.02. (d) Raman spectrum of c-BN NDs showing an evident peak located at 1066 cm^{-1} with a full width half maximum (FWHM) of ~11 cm^{-1}.

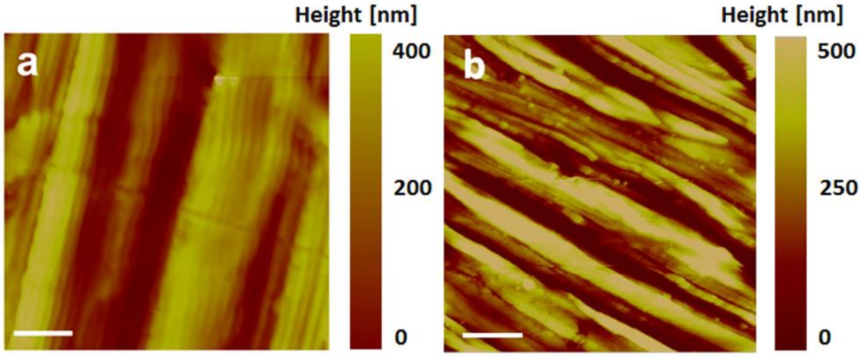

Figure S6. AFM images of the substrates. (a) nickel foil and (b) cobalt foil. The root mean square roughness is 140 and 187 nm for Ni and Co foil substrates, respectively. The scale bars are 10 μm.

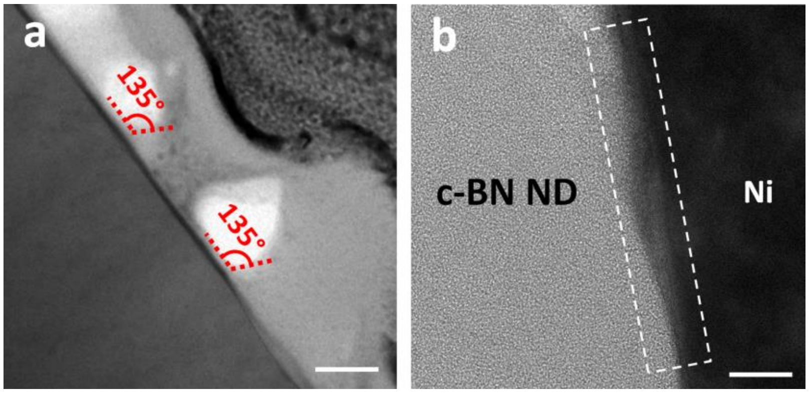

Figure S7. High resolution TEM studies of C-BN ND on Ni substrate. (a) Contact angle measurement of c-BN ND on Ni. According to the cross-sectional bright field TEM image in (a) the contact angle of ~135° was measured for another two NDs with approximately a similar lateral size. (b) The interface structure between the c-BN ND and Ni substrate shows a darker contrast compared to the surrounding area of ND. Both the measured contact angle of above 90° in (a) and different contrasts at the interface region and surroundings in (b) could be indications of strain at the interface between c-BN ND and Ni substrate and subsequent VW growth mode. The scale bars in (a) and (b) are 50 and 5 nm, respectively.

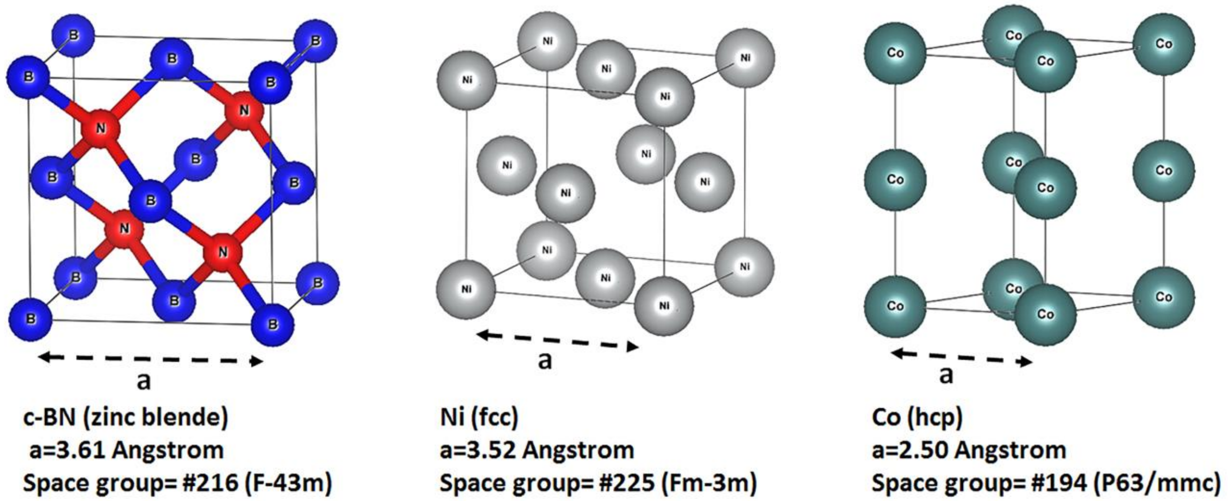

Figure S8. Crystal Structure, lattice constant and space group number of c-BN, Ni and Co.

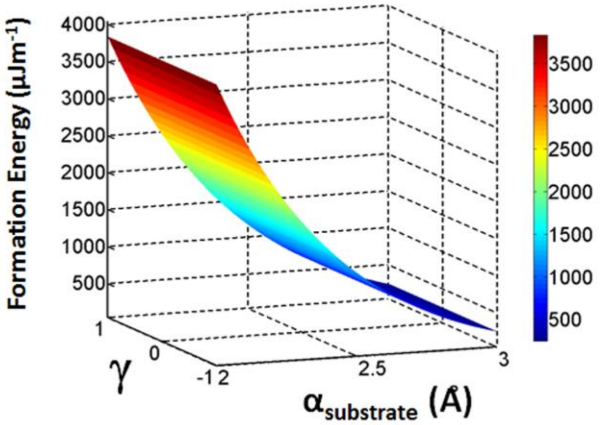

Figure S9. Three-dimensional (3D) plot of the total formation energy of a c-BN ND as a function of system parameter (γ) and substrate unit cell parameter ($\alpha_{substrate}$), assuming the ND is grown on top of a flat metal substrate with a contact angle of 135°.
